# The beneficial role of vitamin B12 in injury induced by ischemia/reperfusion: Beyond scavenging superoxide?

**DOI:** 10.46439/nephrology.2.007

**Published:** 2021

**Authors:** Feng Li

**Affiliations:** Department of Pathology and Laboratory Medicine, The University of North Carolina, Chapel Hill, NC 27599, USA

## Abstract

Vitamin B12 (B12) is required for cellular metabolism and DNA synthesis as a co-enzyme; it also possesses anti-reactive oxygen species (ROS) property as a superoxide scavenger. B12 deficiency has been implicated in multiple diseases such as megaloblastic anemia, and this disease can be effectively cured by supplementation of B12. Multiple studies suggest that B12 also benefits the conditions associated with excess ROS. Recently, we have reported that oral high dose B12 decreases superoxide level and renal injury induced by ischemia/reperfusion in mice. Here, we discuss potential mechanism(s) other than decreasing superoxide by which B12 executes its beneficial effects.

## Vitamin B12

Vitamin B12 is one of the most complex non-protein compounds as described by Dorothy Hodgkin, a *Nobel Prize*-winning chemist, who discovered the molecular crystal structure of B12 [1,2]. Cobalamins (Vitamin B12 derivatives) contain the rare transition metal, cobalt (Co), positioned in the center of a corrin ring and weakly bound to carbon [1]. While cobalamins are synthesized only in certain bacteria and archaea, not in mice or humans [3], they are co-enzymes essential for all life except for plants. For example, in higher vertebrates, methyl-cobalamin and 5’-deoxyadenosyl-cobalamin are essential for the function of methionine synthase and methylmalonyl CoA mutase, respectively [4].

B12 is found naturally in animal products and fortified foods. The daily requirement of B12 intake in humans is less than 5 μg [5]. Once B12 is taken orally, it binds to transcobalamin I (also called haptocorrin or R-protein), secreted by salivary glands to protect B12 from degrading by acid in the stomach. In the duodenum, B12 binds to intrinsic factor (IF), a glycoptotein secreted by the gastric mucosa. B12-IF complex promotes B12 absorption in the intestine [6], and the absorption of dietary B12 requires IF. B12 is released into circulation and transported to various organs after forming a complex with transcobalin II. Via binding to the transcobalamin receptor located on the cell membrane, the complex of B12 and transcobalamin II is internalized into the lysosome [7]. Transcobalamin I is also present in the circulation and binds to B12. However, only livers and kidneys can uptake B12 bound with transcobalamin I [8]. Clearly, intestinal absorption, circulation, cellular uptake, and function of dietary B12 requires complex systems which involve multiple binding proteins, cell surface receptors, lysosomal transfer, and converting enzymes [9]. B12 deficiency caused by defects in any of these systems can lead to megaloblastic anemia, homocysteinemia, methylmalonic acidemia, and other conditions associated with reduced cellular energy metabolism and DNA synthesis [10].

Although these severe clinical conditions are rare today, the subtle subclinical low B12 associated conditions in elder population are getting more attention these days, including neurological disorders, cognitive impairment, inflammatory conditions, and cancer [10,11]. One of the common features of these conditions is increased reactive oxygen species (ROS) associated with aging [12]. B12 is elucidated as a scavenger of superoxide (O_2_^• −^), a major form of ROS in biological system, by Suarez-Moreira et al. [13]. The oxidation state of the cobalt atom is reduced from Co(III) to Co(II) and to Co(I) when B12 is inside of cells. Co(II)balamin is a highly effective intracellular superoxide scavenger with a reaction rate close to that of superoxide dismutases (SOD) [13,14].

## The Role of Vitamin B12 in Renal Ischemia/Reperfusion Injury

We have investigated the therapeutic effects of high-dose dietary B12 in mice with kidney ischemia/reperfuaion injury (IRI), in which the important role of ROS has been well established. Briefly, during the ischemic phase, the absence of oxygen leads to the accumulation of metabolic intermediates. During reperfusion, these metabolic intermediates react with oxygen to produce a sudden increase in oxygen radicals, resulting in the uncontrolled oxidation of cellular components. Furthermore, the impaired mitochondrial electron transfer chain system and nicotinamide adenine dinucleotide phosphate (NADPH) oxidases (NOX) significantly contribute to ROS production during the processes of IRI and repair [15]. We have demonstrated that IRI mice treated with B12 showed near normal renal function and morphology. Further, IRI-induced changes in RNA and protein markers of inflammation, fibrosis, apoptosis, and DNA damage response (DDR) were significantly attenuated by at least 50% compared to those in untreated mice [16]. B12 increased the *Sod* mRNA levels in the kidneys with I/R as well. In vitro, the presence of B12 at 0.3 μM in the culture medium of mouse proximal tubular cells subjected to 3 hr of hypoxia followed by 1 hr of reperfusion showed similar protective effects, including increased cell viability and decreased ROS levels. Importantly, high dose B12 treatment has not caused any observable adverse effects in control mice or cells [16].

Our study suggests the potential application of B12 to the kidney injuries related to excess ROS, especially IRI-induced acute kidney injury (AKI) due to its function of scavenging superoxide. There could be other mechanism(s) by which B12 executes its beneficial effects. Our unpublished data show that B12 increases the survival rate of mouse proximal tubular cell treated with doxorubicin (adriamycin), a topoisomerase II inhibitor. Doxorubicin increases ROS in cardiomyocytes [17] and in freshly isolated rat glomeruli [18]. Thus, it is logical to think that the protective effect of B12 on injury induced by doxorubicin is through its anti-ROS function. However, Morgan et al. showed that there was no correlation between the extent of oxidative stress and cytotoxicity in glomeruli exposed to doxorubicin. They concluded that oxidative stress may not be the primary mechanisms by which doxorubicin induces selective glomerular toxicity [18]. In addition, doxorubicin is shown to alter the expression of genes associate with inflammation [19] and our previous study showed that B12 alter mRNA levels of inflammatory genes [16]. Taken together, it is possible that B12 may also execute its beneficial effects through other mechanisms which are not dependent on anti-oxidative stress such as anti-inflammation.

Autophagy, a highly conserved bulk protein degradation pathway, plays a critical role in regulating homeostasis of proximal tubules in normal physiological condition and integrity in pathological conditions [20–22]. The mice lacking autophagy protein 5 (Agt5, a key regulator of autophagy) specific in proximal tubules have impaired kidney function. The Agt5-null proximal tubule cells show accumulation of p62 (a substrate of autophagy), oxidative stress markers, and deformed mitochondria [20]. While I/R injuries induce autophagy in wild type (WT) mice, mice lacking proximal tubule specific Agt5 are more sensitive to I/R induced injury, including marked increase in serum urea nitrogen and creatinine, and proximal tubule cell apoptosis [20]. While lacking autophagy is harmful to kidneys during I/R, over-activated autophagy in an I/R setting could be detrimental too because activated autophagy flux can also induce cell death [23,24]. Whether activation of autophagy is salutary or harmful perhaps depends on when, how and what degree the signal is activated. Indeed, Chen et al. reported that ELABELA (also known as toddler or apela, a 32-residue peptide) [25,26] and its 11-residue furin-cleaved fragment protected against AKI *via* inhibiting the injury-induced elevation of autophagy influx, inflammation, fibrosis, DDR and apoptosis, indicating that activation of autophagy influx is detrimental [27]. We have not investigated the effects of B12 on the impaired autophagy induced by ischemia (hypoxia)/reperfusion in vivo and/or in vitro. It is interesting to know whether B12 would inhibit the elevation of autophagy influx in I(H)/R setting and protect cell injury associated with impaired autophagy caused by rapamycin, a potent inducer of autophagy [28].

There are more than sixteen different types of cells in the kidneys [29]. There are reports that certain reagents could execute beneficial effects on one type of cells and executes detrimental effects on other types of cells. For example, Mulay et al. reported that nutlin-3a, a chemotherapeutic agent which inhibits murine double minute-2 leading to stabilization and activation of p53, exacerbate IRI by exaggerating the apoptotic response in renal tubular cells. However, nutlin-3a reduced inflammatory responses through apoptosis of dendritic cells in kidneys [30]. In addition, genetically lacking p53 only in proximal tubules prevents IRI due to inhibiting apoptosis [31]. Lacking p53 in leukocytes worsens IRI due to prolonged infiltration resulting from inhibiting apoptosis as well [32]. We reported that B12 decreased mRNA levels of *p53* in the kidneys in the I/R setting. Our unpublished data show that B12 does not alter the mRNA levels of *p53* in mouse macrophage cells undergoing H/R experiment. Taken together, our data indicate that B12 could have different effects on immune cells from those on proximal tubular cells.

It is possible that the beneficial effects of B12 on IRI are through multiple mechanisms. Different cell types and various factors/pathways are implicated in IRI, the precise role of individual cell type and/or factor is unclear. B12 could execute beneficial effects in IRI through inhibiting detrimental factors/pathways in different cell types. Our *in vitro* data clearly shows that B12 inhibits inflammation, fibrosis in kidney proximal tubule cells. It is possible that B12 may inhibit these harmful factors in endothelial cells and/or may promote these harmful factors in monocytes (macrophages) dependent on the stage of the pathological process. Future experimental approach, especially single cell RNA-seq [29], could provide novel information to characterize the complex roles of different cells in IRI and how B12 influence different type of cells.

## Perspectives

Translation of promising laboratory research discovery to human patients has not been always successful. Although B12 has a long history of treating human diseases and general regarded as safe by FDA, more studies involved in both animals and humans are required to elucidate its complex mechanisms and possible application to patients with AKI, especially related to reperfusion injury.
